# Accuracy of interproximal enamel reduction during clear aligner treatment

**DOI:** 10.1186/s40510-020-00329-1

**Published:** 2020-07-28

**Authors:** Maria Elena De Felice, Ludovica Nucci, Adriana Fiori, Carlos Flores-Mir, Letizia Perillo, Vincenzo Grassia

**Affiliations:** 1 Multidisciplinary Department of Medical-Surgical and Dental Specialties, University of Campania Luigi Vanvitelli, Via Luigi de Crecchio 6, 80138 Naples, Italy; 2 Department of Dentistry, Division of Orthodontics, University of Alberta, 5-528 Edmonton Clinic Health Academy, Alberta Canada

**Keywords:** Interproximal enamel reduction, Clear aligner, Stripping, Crowding, Virtual digital planning

## Abstract

**Aim:**

The aim of the present study was to compare the accuracy of the actual space obtained through interproximal enamel reduction (IPR) compared to the amount of IPR planned through the digital setup during clear aligner treatment (CAT).

**Materials and methods:**

A total of 10 clinicians were randomly recruited using the Doctor Locator by Align Technology (California). For each clinician, four consecutive patients treated with CAT and manual stripping were selected for a total of 40 subjects and 80 dental arches. For each patient, the amount of planned IPR and the amount of actual IPR performed were recorded. Each arch was considered individually. For each arch, the mesio-distal tooth measurements were obtained from second to second premolars.

**Results:**

No systematic measurement errors were identified. In 25 cases, stripping was planned and performed in both arches; in 4 cases only in the upper arch and in the remaining 7 cases only in the lower arch. The difference between planned IPR and performed IPR was on average 0.55 mm (SD, 0.67; *P* = 0.022) in the upper arch and 0.82 mm (SD, 0.84; *P* = 0.026) in the lower arch. The accuracy of IPR in the upper arch was estimated to be 44.95% for the upper arch and 37.02% for the lower arch.

**Conclusion:**

Overall, this study showed that the amount of enamel removed in vivo did not correspond with the amount of IPR planned. In most cases, the performed IPR amount was lower than planned. When considering the actual amount in millimeter, these differences may not be considered clinically relevant.

## Introduction

Over the last 20 years, different technological improvements have revolutionized orthodontic treatment planning and execution. Among those, extra-oral scanners have allowed the generation of digital models that can replace clay models for both treatment planning and appliance construction. When associated with increasing patient demands for esthetic and customized approaches, these innovations also have allowed the development of different clear aligner systems as alternatives to the conventional bracket and arch wire orthodontic treatment approaches [1].

The success of clear aligner treatment (CAT) as an alternative orthodontic treatment is based on many potential advantages such as esthetics, increased patient comfort, improved oral hygiene control, and periodontal health compared to fixed appliances [2, 3]. Several related features help the achievement of successful clear aligner treatment outcomes. Among them, the shape and position of the attachment [4] and the CAT material and thickness [5, 6], are noted. Simultaneously, some are related to the patient’s physiology such as bone density [7], tooth crown and root shape [6]. Finally, others depend on operator factors such as treatment planning, monitoring, and the accuracy in performing interproximal enamel reduction (IPR) [8].

Interproximal tooth surface reduction is a common procedure used during orthodontic treatment aimed to reduce mesio-distal tooth size dimensions to address lack of space (mild and moderate crowding), Bolton tooth-size discrepancy, correction of morphologic anomalies, tooth reshaping and management of gingival papilla [9]. Clinically, the most accepted IPR techniques include air-rotor stripping technique with fine tungsten-carbide or diamond burs, hand-piece or contra-angle-mounted diamond-coated disks, and handheld or motor-driven abrasive metal strips [10].

In crowded cases where non-extraction approaches are indicated, IPR can reduce the amount of buccal expansion needed to minimize the periodontal and stability-related risks associated with this tooth movement direction. Also, some studies reported that widened proximal tooth contacts obtained after this procedure can stabilize treatment results [11]. Moreover, correction of anterior crowding with IPR can avoid imperfections known as “black triangles,” due to the presence of ideal gingiva apposition areas that reduce or prevent retrusion of papillae thereby improving esthetic results [12]. Long-term analysis of IPR showed the absence of iatrogenic damage like dental caries, gingival problems, or increased alveolar bone loss [13, 14].

In CAT, IPR is pre-planned during virtual software-based treatment set up. The operator can choose the interproximal areas where to gain space, the amount of enamel to be removed and the stage when to perform movement [15]. Obviously, to get the predicted programmed movements it is important that the amount of IPR actually done is as expected and planned [16]. There is a lack of reliable literature on this subject, especially on the predictability of in vivo stripping. Many related studies focused on the tooth surface after IPR and so far, only two studies have investigated, in vitro, a quantitative evaluation of stripped enamel and its reliability compared to what was supposed to be attained [8, 17]. Hence, the aim of the present study was to evaluate in vivo; the accuracy of space obtained with IPR clinically during CAT compared to the amount of enamel reduction planned through the digital setup.

## Materials and methods

### Subject recruitment

The institutional review board at the University of Campania “Luigi Vanvitelli” granted ethical approval for this prospective study (No. 308 dated 20/51/2019).

The sample size was estimated based on preliminary data. A minimum sample of 39 subjects was needed in order to achieve 80% power, with an alpha of 5% to detect a 0.5 mm difference (SD 0.5 mm).

A total of 10 orthodontists were randomly recruited using the Doctor Locator (DL), an application developed on own website by Align Technology (San Jose, California). Inclusion criteria set for the selection were at least 5 years of experience in CAT; execution of IPR with manual strips; at least 20 patients treated with clear aligner last year. Ten Italian ZIP code have been randomly drawn and entered in the DL application. From the list of providers in that area, the first ten doctors that agreed to participate were included. For each provider, the last four consecutive patients started with clear aligner, and manual stripping was selected for a total of 40 subjects and for a potential total of 80 dental arches.

Patients were recruited according to the following inclusion criteria: adult patients with full permanent dentition, non-extraction orthodontic treatment with CAT, use of composite attachments, treatment plan including IPR (between 0.1 mm and 0.5 mm per tooth), and no visible anomaly of enamel.

### 3D casts and treatment protocol

Before and after stripping, models with silicone impressions were acquired and they were scanned by a 3-dimensional (3D) laser scanner (3Shape, Copenhagen, Denmark). Initial setups were obtained using ClinCheck (Align Technology, San Jose, California), and IPR was planned by each clinician according to the individual patient’s treatment needs. The amount of planned IPR was recorded in an Excel file. Patients were instructed to wear aligners for 22 h per day, except during meals and oral hygiene procedures. Patients were asked to replace aligners on average every 10 days. IPR was performed at the programmed stage according to the virtual treatment staging.

At the scheduled appointment for IPR, separator rings were placed between the teeth for 10 min before the procedure, to make space, to improve visibility and for access to the contact point. Enamel reduction was achieved using single-sided diamond-coated strips (Hopf, Ringleb & Co. GmbH & CIE, Berlin, Germany) and the amount of space obtained was checked with metal gages (Aestetika S.R.L., Terni, TR Italy). Subsequently, polishing strips were used to remove all irregularities. Topical fluoride was also used and left on the reduced teeth for 5 min.

### Measurement protocol

**Fig. 1:**
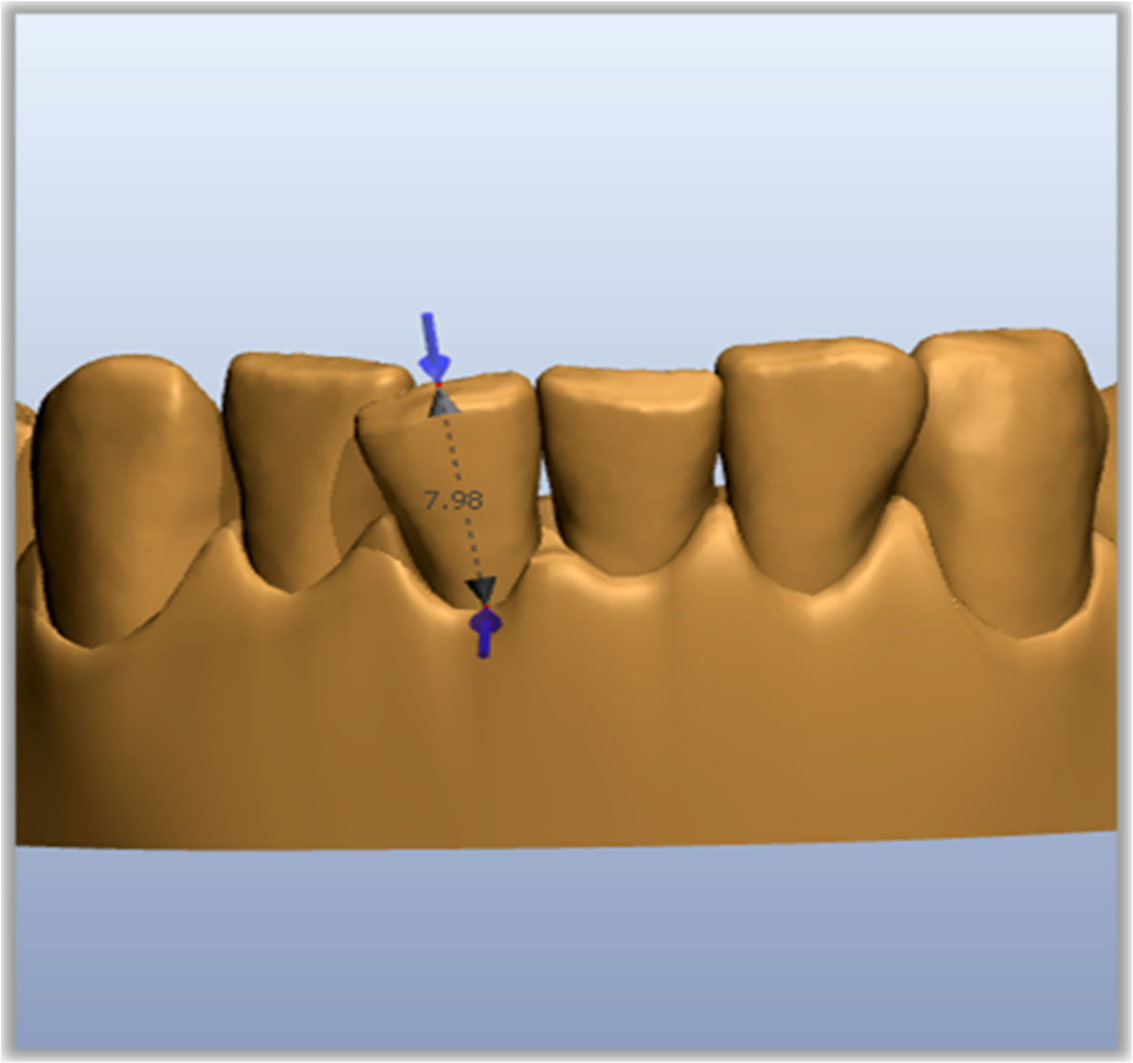
Tooth long axis

**Fig. 2:**
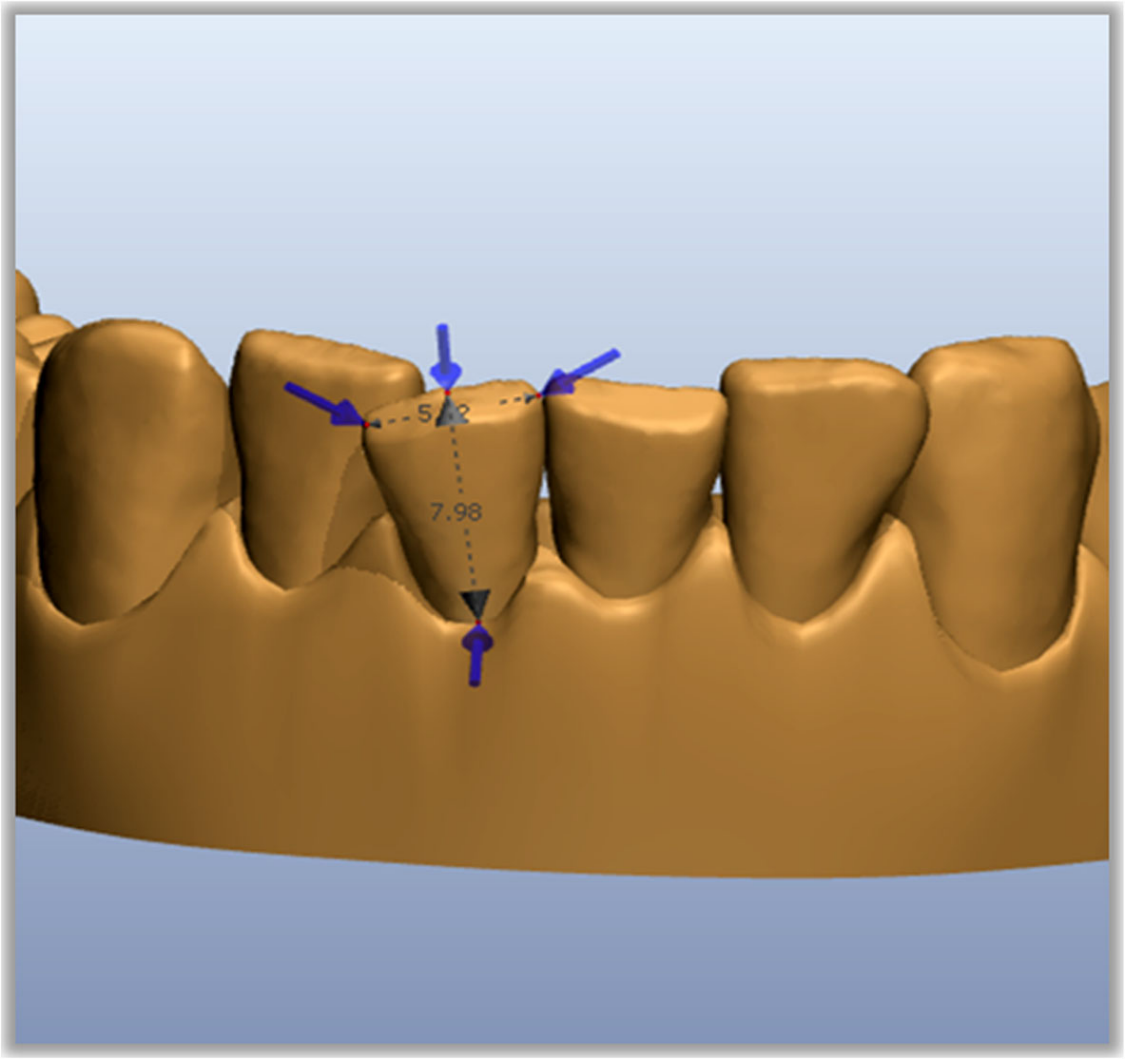
Mesio-distal tooth measurement

**Fig. 3:**
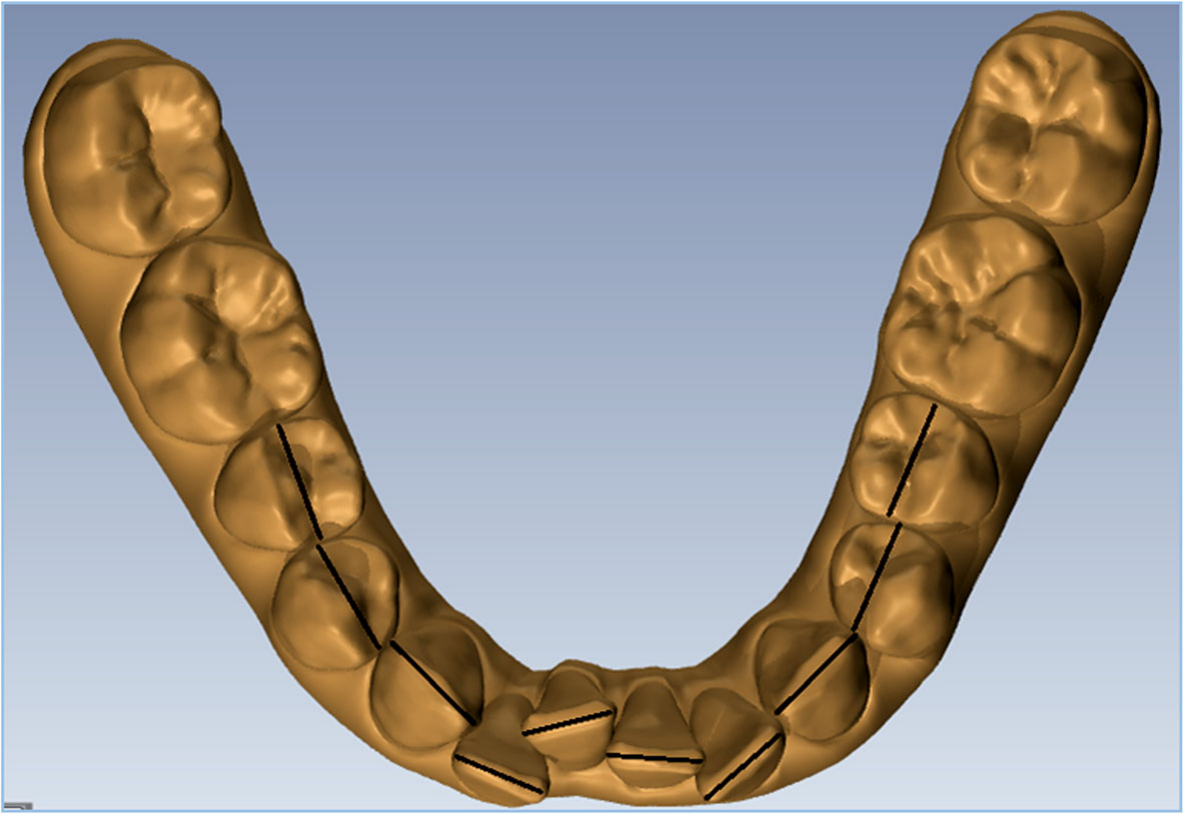
Ʃ35-45 mesio-distal diameters

STL files (Standard Triangulation Language) of the dental casts at the beginning and at the end of aligners planned were exported from ClinCheck and imported into Ortho Analyzer (3Shape, Copenhagen, Denmark). For each patient, we analyzed upper and lower arches before (T0) and after (T1) treatment with planned CAT and IPR. Measurements were performed by the same operator from the second bicuspid to the second bicuspid of each arch. Mesiodistal tooth dimensions were obtained according to the following procedure: as a first step, an operator defined the tooth long axis with a plane, then the software measured the distance from this plane to the farthest points mesially and distally on the tooth (Figs. 1 and 2). Then for each arch, the mesio-distal teeth dimensions were measured from second to second premolar before and after IPR (Fig. 3). Full arch amount of IPR performed was obtained through the difference between the length mesio-distal tooth diameters before and after treatment. The following formula was used to quantify the accuracy of IPR:

$$ \mathrm{IPR}\ \mathrm{accuracy}:\mathrm{percentage}\ \mathrm{of}\ \mathrm{accuracy}\ 100\%\left[\mathrm{IPR}\ \mathrm{planned}-\mathrm{performed}/\mathrm{planned}\kern0.5em \times \kern0.5em 100\%\right] $$

Thus, an index of the accuracy of each movement was obtained: the closer the value to 1, the more precise the IPR was performed by the operator (100% of the prescription). The mean accuracy index, standard deviation, and mean standard error were calculated, and the Student’s *t* test for single samples (*P* < 0.05) was applied in cases in which the accuracy of the IPR was significantly different to 1, i.e., significantly lower than 100% of the prescription.

To evaluate if the amount of anterior crowding could affect the accuracy of IPR, the Little’s irregularity index was calculated for each arch.

### Statistical analysis

For continuous variables, means and standard deviations were calculated. For categorical variables, absolute numbers were reported. Categorical variables were compared with *χ*^2^.

A one-way analysis of variance was used to determine the influence of gender, amount of initial crowding (Little’s irregularity index), and their interactions.

Comparison of continuous variables between time points was made through unpaired *t* tests, while average changes within individual cases were tested through paired *t* tests.

The level of statistical significance was set at *P* < 0.05 for all statistical tests.

### Method of error

A digital caliper (Schieblehre digital 59112; Fino, Bad Bocklet, Germany) was used to verify the accuracy of the virtual measurements comparing them with in vivo mesiodistal premolar widths [8]. Five measurements were performed after 1 month by the same operator to perform error analysis. Measurements were also repeated on eight randomly selected digital models (4 upper and 4 lower arches).

Dahlberg’s *D* was calculated to quantify the measurement error, and Student’s *t* test for paired data to identify any systematic error.

## Results

### Reliability

Measurement method analysis confirmed that there were no systematic measurement errors with any of mesio-distal tooth dimensions before and after treatment as shown in Table 1.

**Table 1:** Error analysis

	Tooth	Mesio-distal tooth diameter T0		Mesio-distal tooth diameter T1	
		***D*** Dahlberg (mm)	Systematic error ***P*** level	***D*** Dahlberg	Systematic error ***P*** level
**Upper arch**	11	0.30	0.12	0.39	0.16
	13	0.29	0.23	0.97	0.12
	15	0.78	0.17	0.27	0.26
	22	0.43	0.12	1.01	0.06
	24	0.67	0.18	0.43	0.14
**Lower arch**	31	0.65	0.09	0.75	0.13
	33	0.47	0.12	0.59	0.14
	35	0.45	0.13	0.86	0.21
	42	0.88	0.25	0.67	0.20
	44	1.11	0.08	0.39	0.11

### Main results

The study group consisted of 40 subjects. In 25 patients, stripping was planned and performed in both arches; in 4 patients only in the upper arch and in the remaining 7 only in the lower arch for an amount of 61 arches selected for our study.

In the upper arch, IPR was planned on average for 1.09 mm (SD: 1.13) of enamel reduction with a maximum value of 3.60 mm and a minimum of 0.30 mm. The IPR programmed in the lower arch was on average more than in the upper (1.43 mm, SD: 1.10) with values from 3.50 to 0.40 mm (Table 2).

**Table 2:** IPR accuracy

	IPR plan (T0)		IPR perf (T1)		Performed—planned IPR (T1-T0)		IPR accuracy	*P* value
	Mean	SD	Mean	SD	Mean	SD		
**Upper arch**	1.09	1.13	0.55	0.64	−0.49	0.53	44.95%	0.022
**Lower arch**	1.43	1.10	0.82	0.84	−0.53	0.66	37.02%	0.026

*P* value < 0.05 was considered as significant

In the upper arch, the mean of IPR performed was 0.55 mm (SD: 0.64) less than programmed (1.09 mm) (Table 2), except for two patients where an excess of enamel of respectively 0.79 and 0.21 mm was removed.

In the lower arch, the amount of enamel removed was on average 0.82 mm (SD, ± 0.84) less than planned (1.43 mm) (Table 2), except for four cases where a reduction of enamel tissue at the end of treatment was recorded as 1.09, 3.31, 1.76, 0.91 mm instead of 0.60, 2.90, and 1, and 0.80 mm with an excess of IPR of 0.49, 0.41, 0.76, and 0.11 mm respectively.

The difference between planned IPR and performed IPR was on average −0.49 mm (SD, 0.53) in the upper arch and −0.53 (SD, 0.66) in the lower arch. A statistically significant difference was found (*P* = 0.022 in upper arch and *P* = 0.026 in the lower arch). Therefore, the accuracy of IPR in the upper arch was estimated to be of 44.95% while this value amounted to 37.02% in the lower arch (Table 2).

The mean of Little’s irregularity index was reduced after treatment, and the difference was statistically significant in both arches (Table 3).

**Table 3:** Little’s irregularity index before and after treatments

	Little’s irregularity index T0		Little’s irregularity index T1		***P*** value
	Mean	SD	Mean	SD	
**Upper arch**	7.26	3.30	2.81	1.72	< 0.001
**Lower arch**	8.13	4.10	2.66	1.57	< 0.001

*P* value < 0.05 was considered as significant

The initial amount of crowding evaluated by Little’s irregularity index, gender, and their interactions were not factors that influenced the accuracy of IPR performed (one-way ANOVA; *P* > 0.05).

## Discussion

The aim of this study was to evaluate the accuracy of IPR during clear aligner treatment. As shown in our outcomes, performed IPR was significantly different from that planned at the beginning of treatment through virtual digital planning. In most of the cases, the amount of actual IPR performed was lower than what was planned. Only in a handful number of cases (2 in the upper arch and 3 in the lower arch) more IPR was done than planned. The clinical impact of these differences is unknown. Such small differences are unlikely to have a major clinical impact in most cases but should be something that clinicians are aware of. If there is an “underperformance” of actual IPR delivered, they may add an “extra” 20-30%” amount of IPR to be done.

IPR plays an important role during CAT in non-extraction approaches to obtain space to align teeth and/or to achieve more long-term alignment stability [16]. So, knowledge of the predictability of this procedure is important to improve treatment outcomes for this technique. This study included only adult patients because they currently represent most of the patients who request orthodontic treatment with CAT and because these patients generally show a better compliance, compared to adolescents, thus reducing a possible source of bias [3].

Different authors have suggested that IPR may be indicated for patients with good oral hygiene and who have either class I arch-length discrepancies with orthognathic profile, minor class II dental malocclusions (particularly in patients who have stopped growing), or Bolton tooth-size discrepancies [3, 8, 18]. In contrast, the major contraindications are as follows: need of space more than 8 mm per arch, active periodontal disease, enamel hypoplasia, tooth hypersensitivity, multiple restorations, round-shaped premolar, young patients with large pulp chambers [14, 19]. It is important to avoid tooth imperfections after IPR which may facilitate an increased risk of caries. Many authors recommend IPR of no more than half the enamel coating’s original thickness to avoid proximity to the dentin [8, 16, 20]. Since teeth show a wide range of morphology variation, some authors have even suggested an enamel thickness measurement taken through radiographs to calculate a more specific reduction [16]. Lapenaite [19] suggests reducing the enamel to a maximum of 0.3 mm per contact point for maxillary incisors, 0.2 mm for mandibular incisors, and 0.6 mm for premolars and molars. This information can be used in clinical orthodontics to decide when to do IPR and be realistic about how much is needed.

Accuracy of IPR performed is a multifactorial issue. The amount of enamel reduction depends on several factors: some are associated with tooth characteristics such as enamel hardness, crown anatomy, tooth position. The results of our study have shown that the amount of anterior crowding measured by the Little’s irregularity index does not influence the accuracy of the IPR performed. Other important factors are the techniques used for enamel removal and the exerted pressure during the enamel reduction procedure, hardness, and particle size of the abrasive, time used to apply it, reliability of space analysis, and operator’s experience [16]. Also, the fact that, when measuring the amount of IPR performed with calibration gages, the pressure added to fit the gages between the contact points creates “non-actual existing” spaces by PDL compression. It can often happen that the measurement of the space obtained is also distorted by the inclination of the gage or in any case by excessive pressure exerted by the operator during its use. These problems can therefore induce errors of both defect and excess. It is also possible that the lower than expected IPR values in our study are due to a fear by the clinician of doing too much IPR or it can be due to the technique used (manual abrasive strips) which are more precise but slower to be completed thereby increasing the need for chair time by the clinician.

In our study, we decided to analyze only the use of handheld abrasive strips. They are manufactured in different sizes and varying thickness and this technique allows for easy access to the interproximal area due to their flexibility and can also be used for finishing or proximal surface recontouring. For these features, they are more reliable and precise and crown morphology deformations or enamel incongruous reduction can be avoided, conditions that could arise with burns. Otherwise their use requires more time, especially in cases where a consistent IPR has been programmed. Johner et al. concluded in an in vitro setting that while testing three different IPR methods that the average amount of enamel reduction obtained was, in general, less than what was intended [8]. However, large variations were observed regardless of the method used. Indeed, they showed that hand-pulled abrasive strips were not as efficient because space obtained after stripping is not as much as expected. The same authors highlighted that the amount of enamel reduction was generally overestimated, especially for intended stripping of 0.2, 0.3, and 0.4 mm [8]. Our results are in agreement with those of Johner et al. that the amount of enamel removed (around 50%) in vivo is generally less than expected, except in a limited number of cases [8]. As shown in Table 2, the amount of enamel removed in vivo was generally less in the lower arch, probably due to greater difficulty for the operator to perform the procedure and due to the tongue obstructing proper access.

The planned IPR in this sample was carried out after analysis of the space and after evaluating the mesio-distal measurements using the dedicated software. This software allows for step-by-step teeth movement programming, moreover, they can virtually separate the teeth and enlarge and rotate them for better visualization. These procedures should allow a much more accurate and simple evaluation of the mesio-distal measurements, compared to the use of a manual caliber or a digital caliber, which are strictly dependent on the operator, on dental cast quality, and on the operator learning curve, in particular, for the use of the manual gage that could be more difficult in cases where the teeth are rotated.

During CAT, the software could plan all IPR required, at the beginning of treatment when the present crowding could make the practice more challenging. It may be suitable to obtain an initial alignment of the teeth before proceeding with the enamel reduction, so it is easier to access the interproximal areas. On the other hand, performing the stripping procedures during alignment could limit teeth movement with a greater periodontal ligament space and therefore greater inherent mobility. So, the perception of enamel reduction could be altered especially with the use of thickness gages.

### Limitations

The main limitation of the present study is that it was not possible to blind the operators who made the measurements given the nature of the study because it would have been easy to recognize and understand the sequence of the digital model. However, care was taken to minimize any selection bias, since all the included subjects were treated consecutively during the considered timespan and were chosen only according to predetermined inclusion criteria.

## Conclusions

Overall, this study showed that the amount of enamel removed in vivo did not correspond with the amount of IPR planned. In most cases, the performed IPR amount was lower than planned.

This study revealed that the accuracy of IPR during clear aligner treatment was of 44.95% in the upper arch and 37.02% in the lower arch. When considering these percentages as an actual amount in millimeter the differences may not be considered clinically relevant.

## Data Availability

Please contact the author for data requests.
